# Is species richness driving intra- and interspecific interactions and temporal activity overlap of a hantavirus host? An experimental test

**DOI:** 10.1371/journal.pone.0188060

**Published:** 2017-11-15

**Authors:** André V. Rubio, Ivan Castro-Arellano, James N. Mills, Rurik List, Rafael Ávila-Flores, Gerardo Suzán

**Affiliations:** 1 Departamento de Etología, Fauna Silvestre y Animales de Laboratorio, Facultad de Medicina Veterinaria y Zootecnia, Universidad Nacional Autónoma de México, Distrito Federal, México; 2 Biology Department, Texas State University, San Marcos, Texas, United States of America; 3 Population Biology, Ecology, and Evolution Program. Emory University, Atlanta, Georgia, United States of America; 4 Área de Investigación en Biología de la Conservación, Universidad Autónoma Metropolitana, Lerma, Estado de México, México; 5 División Académica de Ciencias Biológicas, Universidad Juárez Autónoma de Tabasco, Villahermosa, Tabasco, México; Wageningen Universiteit, NETHERLANDS

## Abstract

High species diversity of the potential animal host community for a zoonotic pathogen may reduce pathogen transmission among the most competent host, a phenomenon called the “dilution effect”, but the mechanisms driving this effect have been little studied. One proposed mechanism is “encounter reduction” where host species of low-competency decrease contact rates between infected and susceptible competent hosts, especially in directly transmitted diseases. We conducted an experiment in outdoor enclosures in northwestern Mexico where we manipulated rodent assemblages to assess the effect of species richness on the frequency of intra- and interspecific interactions and activity patterns of a hantavirus reservoir host (North American deermouse; *Peromyscus maniculatus*). Trials consisted of three treatments of rodent assemblages that differed in species richness, but had equal abundance of deermice; treatment 1 consisted of only deermice, treatment 2 included deermice and one non-competent host species, and treatment 3 included two non-competent host species in addition to deermice. To measure interactions and temporal activity, we strategically deployed foraging stations and infrared cameras. We did not find differences in the frequency of intraspecific interactions of deermice among treatments, but there were significantly more interspecific interactions between deermouse and non-competent hosts in treatment 2 than treatment 3, which is explained by the identity of the non-competent host species. In addition, there were differences in activity patterns between rodent species, and also between deermice from treatment 1 and treatment 2. These results indicate that at least at a small-scale analysis, the co-occurrence with other species in the study area does not influence the frequency of intraspecific interactions of deermice, and that deermice may be changing their activity patterns to avoid a particular non-competent host species (*Dipodomys merriami*). In conclusion, in this deermouse-hantavirus system a potential dilution effect would not be through intraspecific encounter reduction in the most competent hantavirus host. To identify variables of host assemblages that can influence pathogen transmission, we highlight the need to address the identity of species and the composition of assemblages, not only host species richness or diversity.

## Introduction

A topic of growing interest in disease ecology is the relationship between biodiversity and infectious disease transmission [1,2]. A central inquiry has been on the generality and underlying explanations for the so-called “dilution effect.” Given an assemblage containing the potential hosts for a given pathogen in a geographic area, the dilution effect is said to occur when increasing host species richness (the total number of species) or diversity (a metric that considers both richness and relative abundance of species) results in decreasing pathogen prevalence in the primary host species (i.e. the species that maintains and most readily transmits the pathogen). This has been proposed to occur via several potential mechanisms, including decreased primary host population density, reduced frequency of encounters (encounter reduction) between primary host individuals, or reduced survival of primary hosts [3]. The opposite pattern, the ‘‘amplification effect,” occurs when increased host species richness/diversity increases the risk of infection in the primary host, for example, through increased encounters between host individuals [3]. For directly transmitted pathogens (as opposed to arthropod transmitted, for example), assessing the influence of host species diversity on host interactions is of particular interest as co-occurring hosts of low-competency (dilution hosts) may influence contact rates between infected and susceptible competent hosts (amplifying hosts), in turn, increasing or decreasing pathogen transmission. In fact, a recent theoretical study found that interspecific contacts and competition within host communities can be key to determining pathogen transmission, thereby influencing the strength and direction of the diversity-disease relationship [4]. However, despite its relevance, empirical studies regarding the influence of host diversity on intra- and interspecific contact rates of hosts are rare (but see Clay et al. [5], Dizney and Dearing [6]).

The dilution effect is suggested to occur in a variety of host-pathogen systems [7], including rodent-borne hantaviruses (reviewed by Khalil et al. [8]). Hantaviruses (family *Hantaviridae*, genus *Orthohantavirus*) are zoonotic agents responsible for hantavirus pulmonary syndrome (HPS) in the American continent [9]. Transmission between rodent hosts occurs principally by direct contact via saliva or saliva aerosols [10], and aggressive contacts appear to be the main mode of host-to-host transmission of some hantaviruses [11]. In North America, the North American deermouse (*Peromyscus maniculatus*; hereafter deermouse) is the most widely distributed and abundant rodent, and it is recognized as the most competent reservoir host of Sin Nombre virus (SNV) [12], which is the leading cause of HPS in the United States [13]. Several studies in North America have addressed the relationship between small mammal richness/diversity and SNV prevalence [14–16]. For instance, in the Great Basin Desert (Utah) increased levels of rodent species diversity were associated with decreased SNV antibody prevalence [15], as well as decreased encounter rates among deermice, suggesting that encounter reduction may be the mechanism that drives the dilution effect in this host-hantavirus system [5]. However, correlative studies such as this are limited in that they cannot control for differences in the density and diversity of the rodent communities, or other environmental factors (such as habitat structure) that may modulate encounter rates [5]. Manipulative experiments are useful in separating alternative explanations of these types of associations between rodent interactions and species diversity.

Manipulative field experiments have been conducted to analyze the influence of small-mammal diversity on hantavirus infection prevalence [17]. However, to our knowledge there have been no studies that manipulate species richness to assess its influence on the frequency of intraspecific interactions of a hantavirus reservoir. Furthermore, interaction rates can be influenced by several variables including temporal niche partitioning and social behaviors (i.e., avoidance and agonistic) among species [4]. Thus, those variables mentioned above need to be considered as an integral part of an inquiry into interaction rates among individuals. We conducted a manipulative experiment in outdoor enclosures to test for differences in frequencies of intra- and interspecific interactions of deermice among three treatments that differed in rodent species richness. We also included in our analyses comparisons of temporal activity patterns, temporal niche overlap, and behavioral interactions (i.e. aggressive, passive, and avoidance) between treatments and species.

Our experiment was aimed to test the following predictions: (a) An increase in rodent species richness will decrease the intraspecific contact rates among deermice, and (b) An increase in rodent species richness or presence of a particular non-competent host species will influence the temporal activity of deermice. Findings derived from these experiments may have implications for transmission of pathogens in the context of the biodiversity-disease relationship.

## Materials and methods

### Study site and enclosures

The study was carried out on the Nature Conservancy´s El Uno Ecological Reserve (Private Lands Program, Mexico; 30°50'17"N, 108°25'36"W), within the Janos Biosphere Reserve, in northwestern Chihuahua, 75 km south of the United States-Mexico border. A mosaic of desert grasslands and shrublands dominates this arid landscape, with interspersed patches of riparian vegetation, agricultural lands, and human settlements. There is evidence of hantavirus infection in deermice in this area [18,19].

To conduct an experimental study in the field, we used outdoor enclosures, which are environments that approximate natural field conditions, but also allowed us to manipulate and control rodent assemblages. These types of enclosures have been used successfully in investigations on rodent ecology [20,21], including ecological studies on rodent-borne hantaviruses [22,23].

**Fig 1:**
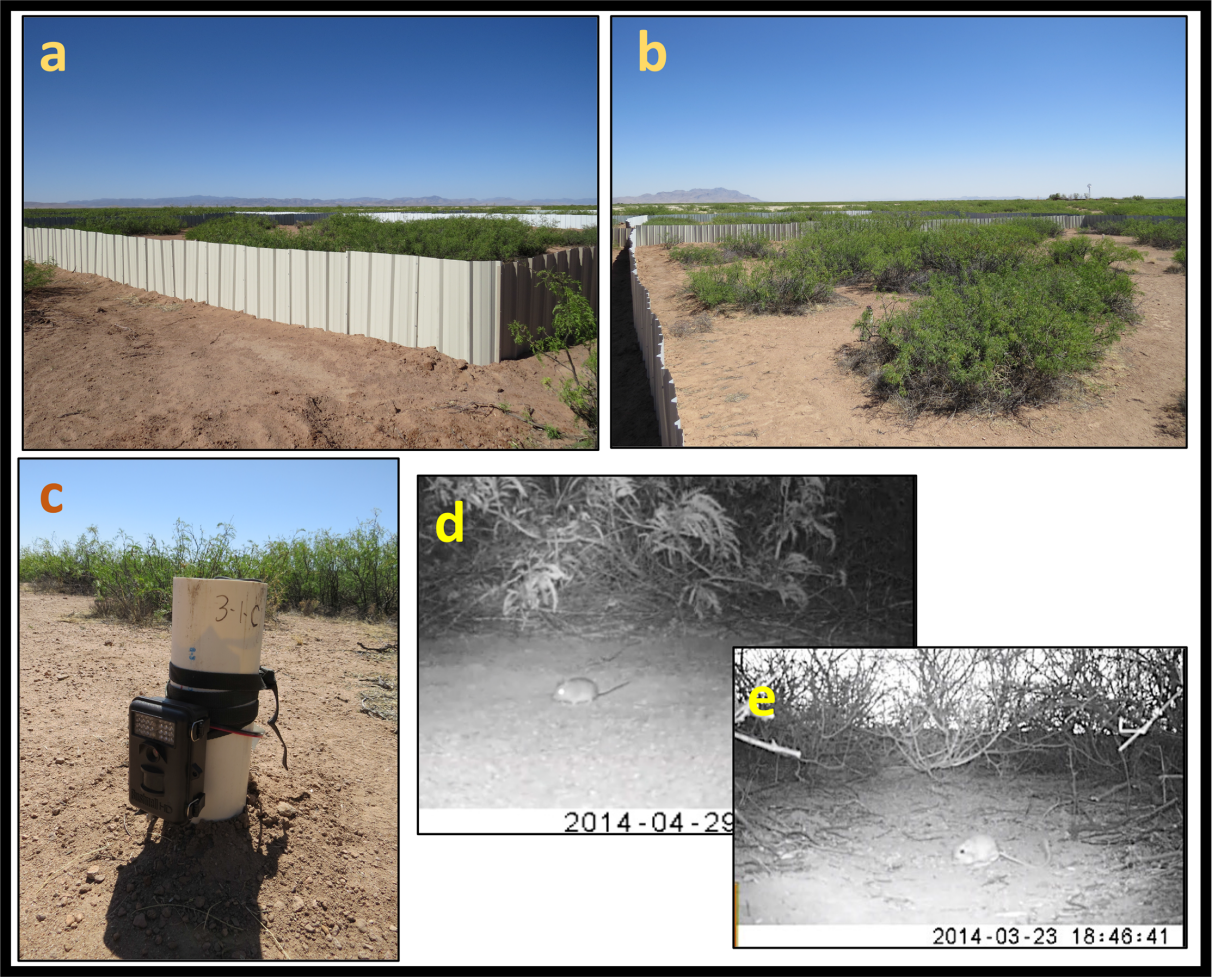
Enclosures and photos from video records. (a & b) Enclosures consisted of sheet metal walls, (c) a camera trap placed within an enclosure for video recording, (d) photo of a deermouse in the field, and (e) photo of a kangaroo rat in the field.

The experiment was run in three 0.1-ha enclosures (30 m x 30 m) (Fig 1) situated in mesquite (*Prosopis glandulosa*) shrubland, the main habitat of deermice in the area [19]. Each enclosure consisted of sheet metal walls, which extended approximately 1 m above ground and 0.6 m underground. Within each enclosure, shrubs covered 300–315 m^2^ (33–35% of their total area), and spatial distribution of shrubs was similar among enclosures. To impede escapes from the enclosures, all vegetation within 1 m of the enclosure walls (inside and outside) was removed. Within each enclosure we observed several natural rodent burrows. In addition, we placed eight PVC pipes (5 cm diameter and 40 cm tall) in each enclosure evenly spaced and buried (at a 45-degree angle) to serve as shelters. Prior to conducing the experiment, all rodents within the enclosures were removed using Sherman live-capture traps (8 x 8 x 23 cm; H.B. Sherman traps, Tallahassee, Florida).

### Study design

**Fig 2:**
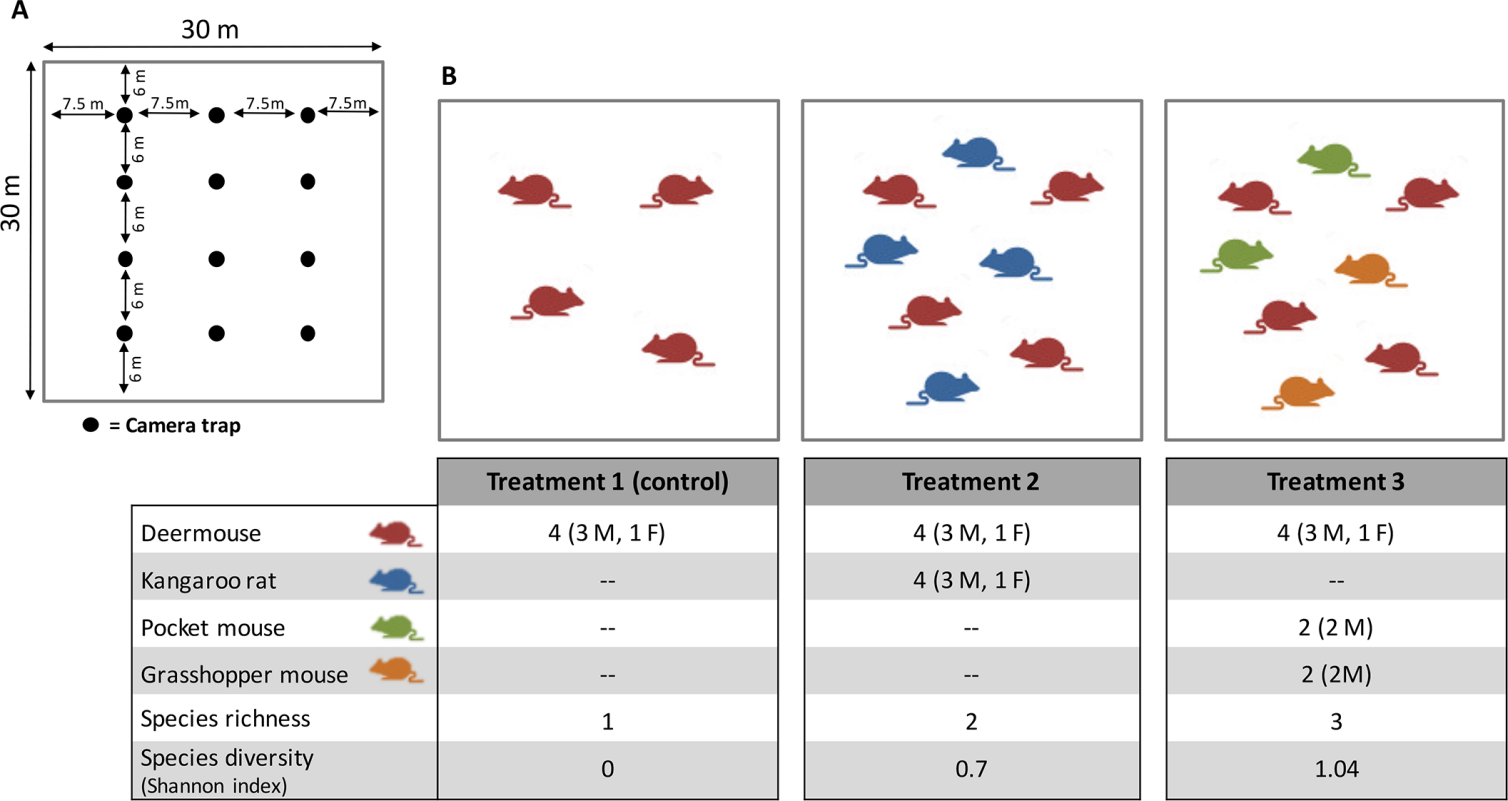
Diagrams of enclosures and experimental design. (A) Diagram of an enclosure with the camera trap array. (B) Diagram of the experimental design. Each column has the number of individuals (M = male, F = female), species richness, and species diversity of each treatment.

The experiment included three treatments (T1: control group, T2 and T3), which simulated three different rodent assemblages. Each treatment had the same number of deermice (focal species), but different species richness and relative abundance (Fig 2), to identify the effect of species richness *per se*, rather than the effects of varying population densities of deermice. Three additional rodent species were selected for the study because they are the most abundant species in the local mesquite shrublands [19], and are considered non-competent hosts as they may become incidentally infected, but are not known to maintain or transmit hantaviruses [12,24,25]: the Merriam's kangaroo rat (*Dipodomys merriami*, hereafter kangaroo rat), which is the dominant species in this habitat, the desert pocket mouse (*Chaetodipus penicillatus*, hereafter pocket mouse), and the Chihuahuan grasshopper mouse (*Onychomys arenicola*, hereafter grasshopper mouse). The first two belong to the family Heteromyidae, while the latter and the deermouse belong to the family Cricetidae. The four species used in the experiment represent nearly 85% of the total abundance of the rodent assemblage in the local mesquite shrublands [19].

Because male deermice are responsible for most SNV transmission in wild populations [26], we included in each treatment a higher number of males than females (Fig 2). Kangaroo rats had the same sex ratio as deermice, whereas the other two species were represented only by males (Fig 2). In all cases, males were preferred in our experiments considering that they might exhibit stronger territorial behaviors than females. The final number of experimental individuals was established to have a similar proportion of males from other species against the focal species (deermice). Although population densities of some rodent species in the study area are lower than in the enclosures, we included higher numbers of individuals to allow statistical analyses. Our previous data [19] indicate that in a 0.1-ha area, deermice usually have an abundance of 1–2 individuals, and the total rodent abundance can reach 6–7 individuals. Thus, considering fluctuations typically observed in rodent populations, the total density of individuals used in the enclosures is within the limits of natural variation for this area. All individuals used in each enclosure were from different capture sites (see below for more details), to minimize the probability of prior contacts among individuals before the experiment.

From March 9^th^ to May 13^th^, 2014, we conducted six experimental trials (repetitions) for each treatment. Each trial was considered independent as we used different rodent individuals for each trial. A trial started when we introduced all individuals into the enclosures simultaneously for all three treatments and lasted six nights. Individuals were assigned to treatments randomly. The first two nights were considered an adaptation period and data were collected the following four nights. Upon completion of a trial, all rodents were captured and removed from the enclosures (using Sherman traps) and released at their site of original capture in the wild. In the following trial, new individuals were introduced into the enclosures and we alternated the enclosures housing for each treatment to avoid possible bias due to eventual differences between enclosures. Therefore, each enclosure was used for two trials of each treatment.

### Rodent collection

Prior to each experimental trial, we placed transects in several sites within mesquite shrubland, which consisted of 20–30 Sherman traps 10 m apart, to collect rodents for the experiment. Distances between transects were at least 500 m. Once trapped, adult individuals of study species were selected, based on body weight and size. The selected males had their testes either in scrotal or abdominal position (but were placed in the same proportion into each enclosure). The selected females were in non-breeding condition (closed vagina) and had no evidence of pregnancy. Individuals were ear tagged or pelage was uniquely shaved in a small area of the body and transferred to individual wooden cages for a maximum of five days. Water and food were provided until released into the enclosures.

### Ethics statement

All procedures for trapping and handling rodents followed the guidelines of the American Society of Mammalogists for the use of wild mammals in research [27]. The protocol was approved by the Committee on the Ethics of Animal Experiments of the Veterinary School, Universidad Nacional Autónoma de México (CICUAE-FMVZ/UNAM), and by the Secretariat of Environment and Natural Resources of Mexico (SEMARNAT, Permit FAUT-0250).

### Temporal activity and rodent interactions

In each enclosure, we placed 12 foraging stations in a 4 x 3 grid set at 6 and 7.5 m intervals (Fig 2). Each foraging station contained ~ 30 g of rolled oats with vanilla extract partially covered by soil and located 50 cm from a mesquite bush. In front of, and 1.7 m from each foraging station, we installed one infrared camera trap (Bushnell Trophy Cam 119537C, Bushnell Optics, Overland Park, Kansas) mounted 10 cm above the ground on a PVC pole (Fig 1). Camera traps have been used to monitor small mammals, as they can readily detect and reliably identify small mammals to species [28,29]. Pilot trials of camera performance were conducted in the laboratory and in the field to test their effectiveness in recording rodent presence and movements (Fig 1). Based on our trapping surveys with Sherman traps, we confirmed that the bait used in the foraging station attracts all four species. Cameras recorded a 30-sec video once an individual visited the foraging stations (Fig 1), and continued recording as long as at least one individual stayed in front of the sensor. Each camera recorded an area of ~ 1.5 m^2^ (total area covered by cameras was approximately 18 m^2^ per enclosure). This monitoring approach did not allow reliable identification of specific individuals in all recordings because of the fast movement of rodents. Therefore, all analyses were to species, not individual. Each video from the same camera that recorded the same species after at least a one-min interval was considered an independent visit to the station. For each treatment and trial, we quantified the number and time of species-specific visits to the foraging stations and the number of intra- and interspecific interactions. Rodent behavioral interactions were classified into three categories: 1. Aggressive (fighting or chasing), 2. Passive (individuals sharing the foraging station without any aggressive action), and 3. Avoidance (one or more individuals avoid another by leaving the station). We defined a minimum interval of 10 min between videos for considering each interaction as independent.

### Data analysis

We compared the number of deermouse visits to the foraging stations and number of intra- and interspecific interactions of deermice among treatments using generalized linear models (GLM) with quasi-Poisson distribution and log-link function to account for overdispersion in response variables. For the analysis of intraspecific interactions, we pooled all three types of behavioral interactions but also analyzed aggressive encounters separately. For analysis of interspecific interactions of deermice, first, we compared the absolute number of interactions among treatments and, in a second analysis, we compared the number of interspecific interactions of deermice among the three non-competent host species standardized by the number of individuals of each species, because non-competent host species vary in their abundances (Fig 2). In this later comparison, we used the Kruskal–Wallis test because standardized number of interactions were non-normally distributed. We did not include in the analyses the effects of enclosures because the enclosure housings were alternated for each treatment. Neither food provision nor number of artificial shelters per rodent individual were included in the analyses because we observed that rodents used natural burrows and also fed from other natural resources (e.g. mesquite seeds). We performed these analyses with R software (R Development Core Team 2017).

Pairwise comparisons of temporal activity patterns of deermice among experimental groups were evaluated with Watson's two-sample U^2^ test, which is a nonparametric goodness-of-fit test developed for cyclic distributions [30]. This test was also used for pairwise evaluations of interspecific differences in activity patterns among all species. We performed these analyses with Oriana 4.02 [31].

We used Monte Carlo simulations to evaluate assemblage-wide temporal niche overlap among all species. The basis for analysis consisted of a species-by-time interval matrix that was populated with the number of records for each species at each 30-min and 1-h interval. Overlap was quantified as the average of all pairwise overlap values calculated via the Czechanowski index [32]. This empirical index is compared against the null distributions of overlaps generated by the simulation algorithm. Temporal segregation (i.e., less overlap than expected by chance) is indicated by an unusually small overlap while compared to random assemblages generated by simulations, and temporal coincidence (i.e., more overlap than expected by chance) is designated by an unusually large overlap when contrasted against those simulated assemblages. Null distributions of overlap values were generated using a randomization algorithm (Rosario) that was designed specifically for temporal data [33]. Rosario maintains the empirical structure of the data while creating the simulated scenarios in each iteration. Rosario not only maintains temporal autocorrelation of temporal data but also creates a more biologically meaningful null space and it is less prone to Type I errors. Simulations using Rosario were conducted with the Time Overlap program (freely available at: http://hydrodictyon.eeb.uconn.edu/people/willig/Research/activity%20pattern.html). Detailed information and bench tests for this algorithm are available elsewhere [33].

Since temporal activity may change through time because of day length, and the experiment was conducted from March to May, first, we conducted all analyses of temporal activity in two data sets separately; the first data set included trials from March 9^th^ to March 26t^h^ (late winter, three trials of each treatment) and the second included trials from April 26^th^ to May 13^th^ (spring, three trials of each treatment). Second, both data sets were pooled and analyzed together.

## Results

**Fig 3:**
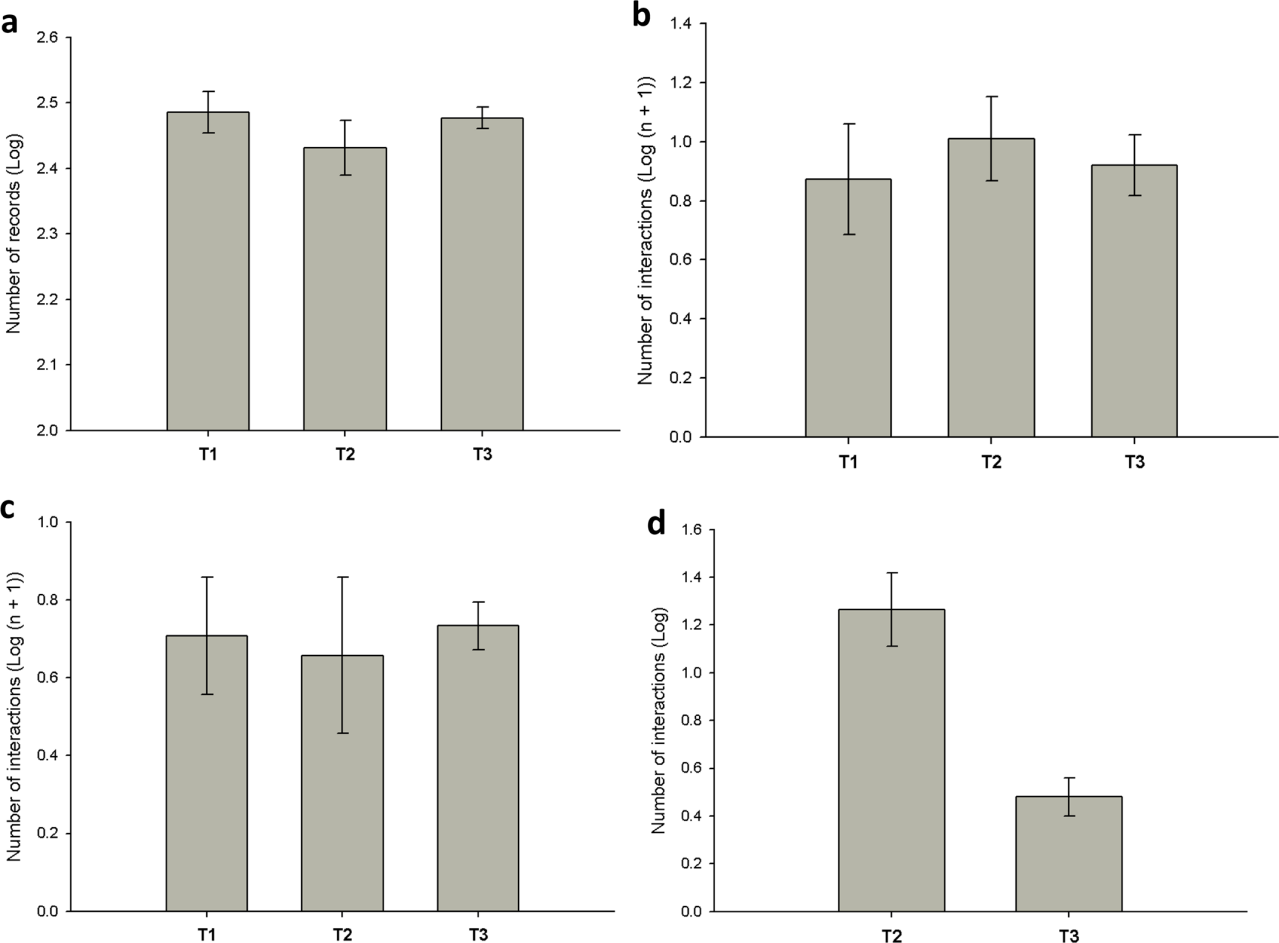
Mean (± standard error) of deermouse visits to the foraging stations and mean (± standard error) number of intra- and interspecific interactions of deermice (DM) among treatments (T). (a) Number of records of DM at foraging stations; (b) number of intraspecific interactions of DM; (c) number of aggressive interactions between DM; (d) number of interspecific interactions of DM.

**Fig 4:**
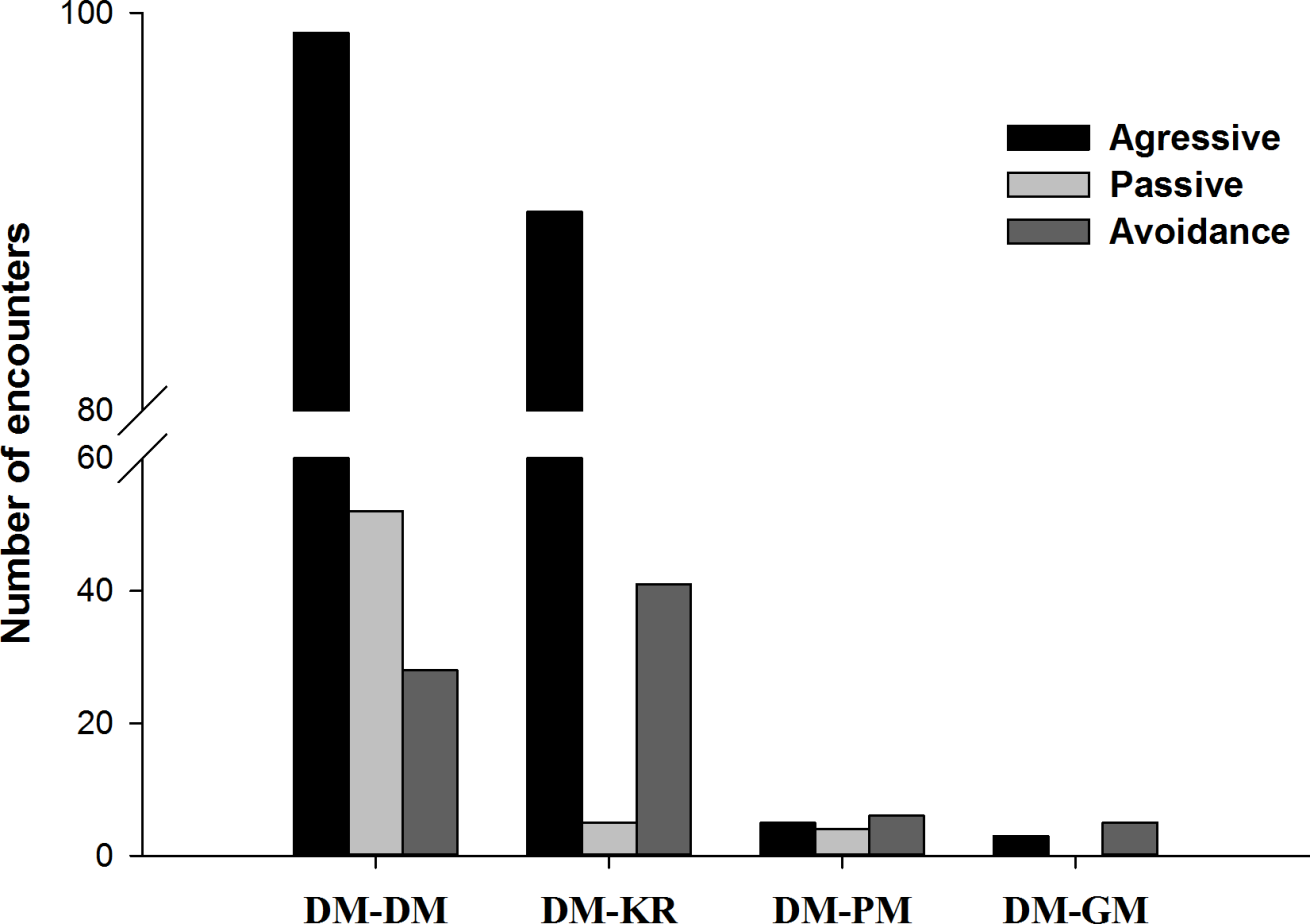
Number of encounters by type of interaction. DM (deermouse); KR (Kangaroo rat); PM (pocket mouse); GM (grasshopper mouse).

We tallied 11,244 video records: 6,251 for deermice, 3,821 for kangaroo rats, 555 for pocket mice, 441 for grasshopper mice, and 176 for two species at the same time. In general, deermice were solitary foragers; 95% of the videos recorded one individual. There were no differences among treatments in the number of deermice records at the foraging stations (Table 1; Fig 3). We recorded 179 videos of intraspecific interactions of deermice (S1 Dataset), and aggressive was the most common type of interaction (Fig 4). There were no differences in the numbers of all intraspecific interactions of deermice among treatments (Table 2; Fig 3) and there were no differences when analyzing only aggressive behaviors (Table 3; Fig 3). We recorded 164 interspecific interactions of deermice (S1 Dataset). The numbers of these interspecific interactions differed between treatments 2 and 3 (Table 4; Fig 3), where kangaroo rats had significantly more interactions with deermice (standardized median = 5.88), compared to grasshopper mice (standardized median = 0.5) and pocket mice (standardized median = 1.25) (H = 12.77; P = 0.002). Most interactions between deermice and kangaroo rats were aggressive (Fig 4). In all aggressive interactions kangaroo rats attacked and chased deermice. For all avoidance interactions between these species, deermice avoided kangaroo rats. Interactions of deermice with the other species were few and mainly aggressive or avoidance (Fig 4). For all aggressive interactions between deermice and grasshopper mice or pocket mice, deermice attack and chase the other species.

**Table 1:** GLM (quasi-Poisson distribution model) results. Deermice visit to the foraging stations. Bold font indicates significant *P* values.

	Estimate	SE	*t* value	*P*
(Intercept)	5.921	0.074	79.981	**<0.001**
T2	-0.051	0.106	-0.498	0.626
T3	-0.107	0.108	-0.990	0.338

(Dispersion parameter for quasi-Poisson family taken to be 12.253)

Null deviance: 191.20 on 17 degrees of freedom

Residual deviance: 179.17 on 15 degrees of freedom

**Table 2:** GLM (quasi-Poisson distribution model) results. Intraspecific interactions of deermice. Bold font indicates significant *P* values.

	Estimate	SE	*t* value	*P*
(Intercept)	2.216	0.304	7.287	**<0.001**
T2	0.297	0.401	0.739	0.471
T3	-0.095	0.441	-0.216	0.832

(Dispersion parameter for quasi-Poisson family taken to be 5.084)

Null deviance: 88.673 on 17 degrees of freedom

Residual deviance: 83.444 on 15 degrees of freedom

**Table 3:** GLM (quasi-Poisson distribution model) results. Aggressive interactions between deermice. Bold font indicates significant *P* values.

	Estimate	SE	*t* value	*P*
(Intercept)	1.674	0.370	4.524	**<0.001**
T2	0.198	0.499	0.396	0.698
T3	-0.134	0.542	-0.247	0.809

(Dispersion parameter for quasi-Poisson family taken to be 4.381)

Null deviance: 68.117 on 17 degrees of freedom

Residual deviance: 66.257 on 15 degrees of freedom

**Table 4:** GLM (quasi-Poisson distribution model) results. Interspecific interactions of deermice among treatments. Bold font indicates significant *P* values.

	Estimate	SE	*t* value	*P*
(Intercept)	3.178	0.204	15.565	**<0.001**
T3	-1.974	0.585	-3.376	**0.007**

(Dispersion parameter for quasi-Poisson family taken to be 6.003)

Null deviance: 170.69 on 11 degrees of freedom

Residual deviance: 64.96 on 10 degrees of freedom

**Fig 5:**
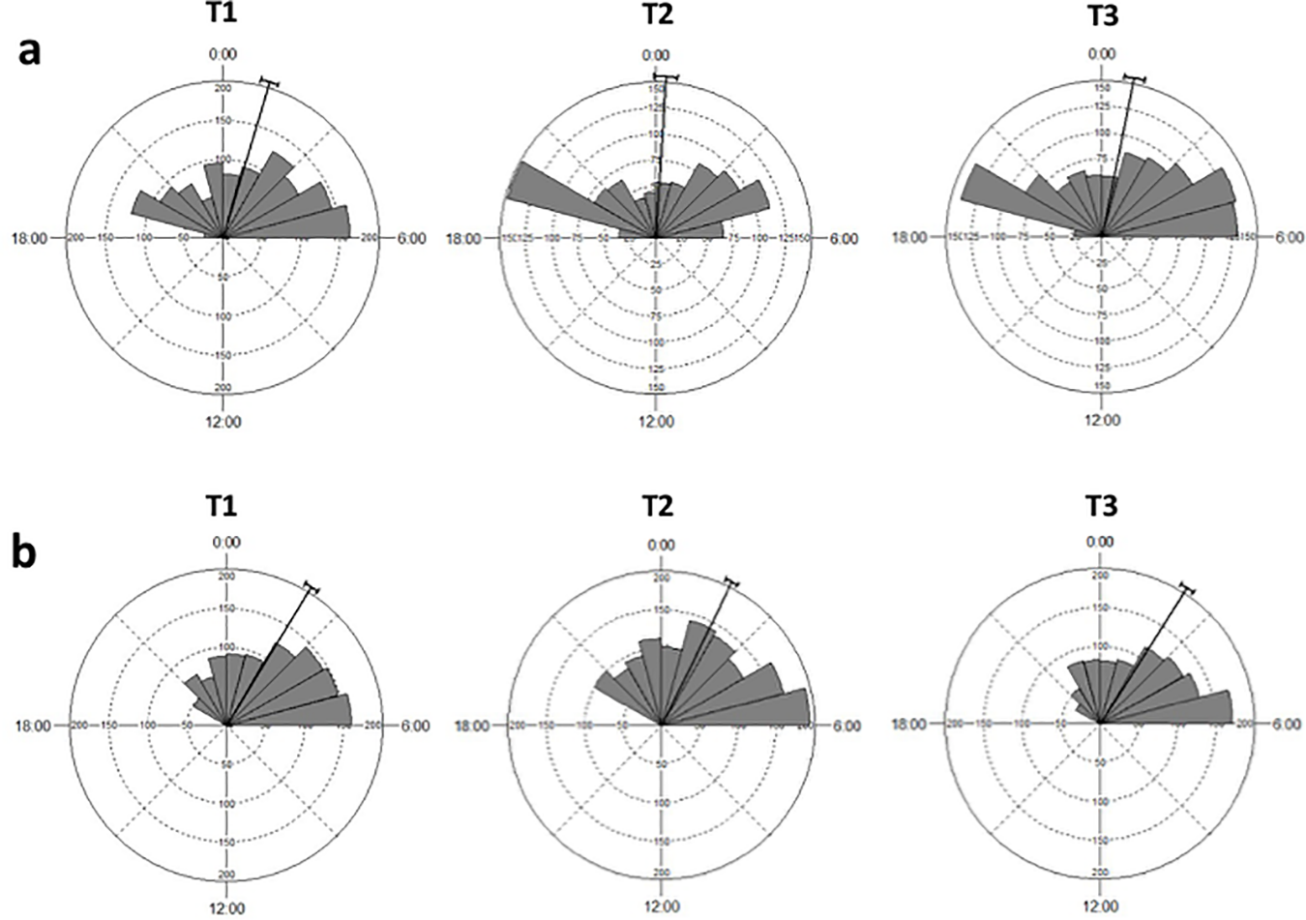
Diel activity patterns of deermice in each treatment (T). (a) samples from late winter, and (b) samples from spring. Bars indicate the proportion of independent records taken at that time of the day. Tick lines represent the mean vector and its circular standard deviation.

**Fig 6:**
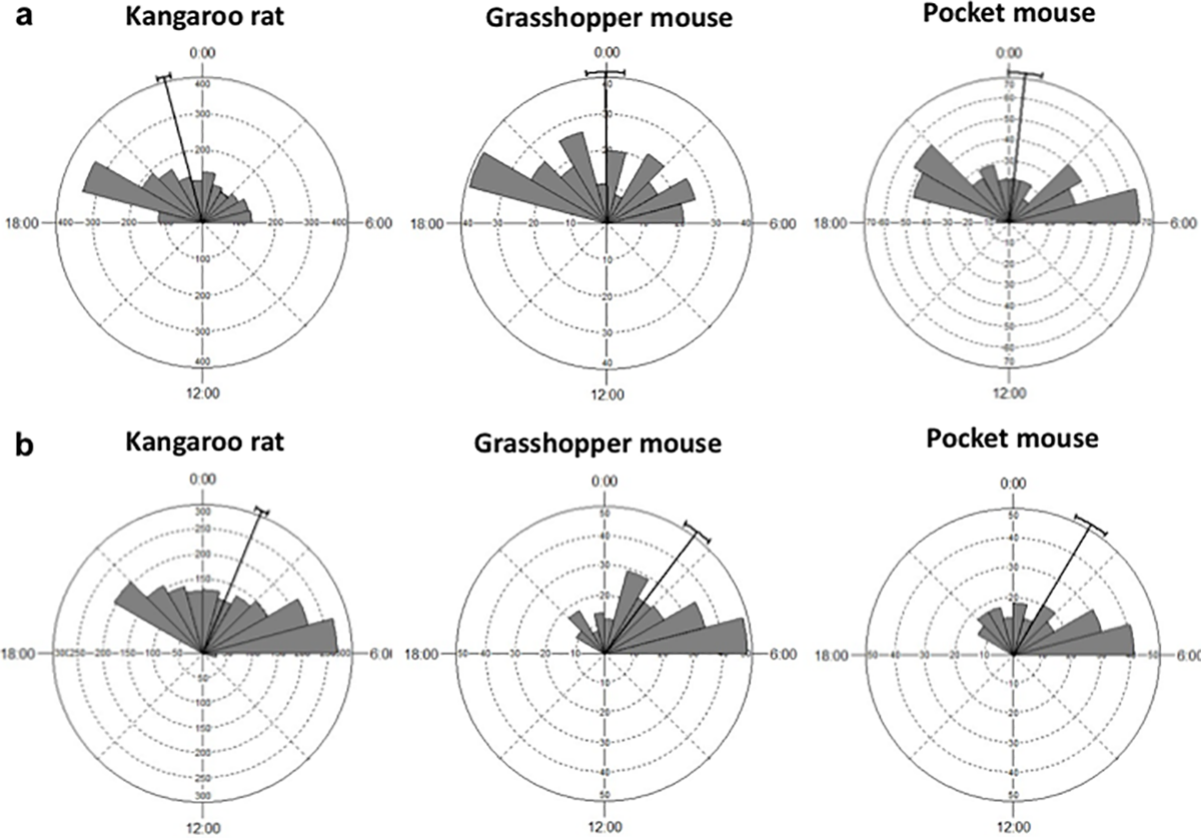
Diel activity patterns of non-competent hosts species. (a) samples from late winter, and (b) samples from spring. Bars indicate the proportion of independent records taken at that time of the day. Tick lines represent the mean vector and its circular standard deviation.

Regarding temporal activity patterns, all species were active from 18:40 h to 06:40 h (Figs 5 and 6; S1 Dataset). Differences in activity patterns between late winter and spring were found for all species (Table 5). Activity patterns of deermice of treatment 1 differed significantly from those of treatment 2 in both seasons, while no differences of deermice activity were found between treatments 1 and 3 (Tables 6 and 7). There were also differences of deermice activity between treatments 2 and 3 only in spring (Tables 6 and 7). When data from both season were pooled, all significant results disappeared (S1 Table). Differences in activity patterns between pairs of species were found between deermice and all non-host species in late winter, while in spring there were significant differences between deermice and kangaroo rats and pocket mice (Tables 6 and 7). Difference between non-host species were also observed (Tables 6 and 7). Assemblage-wide activity overlap was highly consistent among species. For all cases, there was a larger assemblage-wide temporal overlap when compared to the random expectation (Table 8 and S2 Table).

**Table 5:** Mean vector (μ) and circular standard deviation of activity patterns of each species in each treatment (T). Data shown are the time of the day and degrees (in parentheses). Watson's U^2^ test was used for comparisons of activity patterns between seasons. Bold font indicates significant *P* values. DM (deermouse); KR (Kangaroo rat); PM (pocket mouse); GM (grasshopper mouse).

	Late Winter			Spring				
Species	n	μ	SD	n	μ	SD	U^2^	*P*
DM (T1)	1195	01:06 (16,604°)	03:38 (54,741°)	1047	02:05 (31,408°)	02:50 (42,515°)	1.217	**<0.001**
DM (T2)	866	00:14 (3,505°)	03:54 (58,702°)	1252	01:44 (26,051°)	02:56 (44,21°)	2.203	**<0.001**
DM (T3)	1034	00:46 (11,541°)	03:49 (57,392°)	965	02:10 (32,513°)	02:46 (41,593°)	1.907	**<0.001**
KR (T2)	1799	23:00 (345,117°)	03:42 (55,535°)	1667	01:28 (22,153°)	03:21 (50,337°)	4.319	**<0.001**
GM (T3)	230	23:57 (359,284°)	03:38 (54,689°)	219	02:27 (36,981°)	02:49 (42,25°)	0.795	**<0.001**
PM (T3)	374	00:24 (6,032°)	04:01 (60,389°)	202	02:02 (30,571°)	03:10 (47,722°)	0.686	**<0.001**

**Table 6:** Pairwise comparisons (Watson's U^2^ test) of activity patterns among rodents in late winter. Values above the diagonal correspond to P values. Values below the diagonal correspond to U^2^ statistic values. Significant results in bold.

Deermouse^1^	T1	T2	T3	
**T1**	-	**< 0.005**	0.2 > p > 0.1	
**T2**	0.317	-	0.5 > p > 0.2	
**T3**	0.13	0.099	-	
**Interspecies**	**Deermouse**	**Kangaroo rat**	**Pocket mouse**	**Grasshopper mouse**
**Deermouse**	-	**< 0.001**	**< 0.001**	**< 0.002**
**Kangaroo rat**	1.323	-	**< 0.005**	**< 0.05**
**Pocket mouse**	0.42	0.213	-	0.1 > p > 0.05
**Grasshopper mouse**	0.352	0.212	0.168	-

^1^ Comparison of deermice between treatments (T).

**Table 7:** Pairwise comparisons (Watson's U^2^ test) of activity patterns among rodents in spring. Values above the diagonal correspond to P values. Values below the diagonal correspond to U^2^ statistic values. Significant results in bold.

Deermouse^1^	T1	T2	T3	
**T1**	-	**< 0.05**	> 0.5	
**T2**	0.228	-	**< 0.02**	
**T3**	0.044	0.25	-	
**Interspecies**	**Deermouse**	**Kangaroo rat**	**Pocket mouse**	**Grasshopper mouse**
**Deermouse**	-	**< 0.001**	**< 0.02**	0.5 > p > 0.2
**Kangaroo rat**	1.178	-	0.2 > p > 0.1	**< 0.001**
**Pocket mouse**	0.252	0.122	-	0.1 > p > 0.05
**Grasshopper mouse**	0.093	0.606	0.145	-

^1^ Comparison of deermice between treatments (T).

**Table 8:** Results of ROSARIO algorithm null model analyses of temporal niche overlap (both seasons combined). Overlap was quantified as the average of all pair-wise overlap values calculated via the Czechanowski index, using the numbers of records for each species at two time intervals (30 min and 1 h). P-values are two-tailed probabilities of finding non-random assemblage-wide temporal niche overlap. Tail (T) indicates that empirical overlap occurs on the right-hand (R) side of the simulated distribution. Values in the extreme right indicate coincident activity patterns. Values on the extreme left would have indicated segregated activities. Significant results in bold. Results of temporal niche overlap separated by season are provided in S2 Table.

		Rosario			
Tested group	Observed overlap	Simulation overlap	SD	P-value	T
*30-min interval*					
Treatment 3^1^	0.86	0.71	0.03	**< 0.001**	R
All species^2^	0.86	0.74	0.02	**< 0.001**	R
*1-h interval*					
Treatment 3^1^	0.91	0.70	0.04	**< 0.001**	R
All species^2^	0.89	0.73	0.02	**< 0.001**	R

^1^All three species from treatment 3.

^2^All four species used in the experiment. Data of deermice from all treatments were pooled.

## Discussion

### Host behaviors and the effect of species richness on interactions

Our results indicate that deermice usually have a solitary foraging behavior, an observation consistent with a recent study conducted in Utah [34]. Both studies also found that most intraspecific interactions among deermice are aggressive behaviors, which are suggested to be the main transmission route for some hantaviruses, including SNV [11, 35]. Our experimental approach did not evince differences in the number of intraspecific interactions between deermice in environments with greater rodent species richness. In contrast, Clay et al. [5] found a negative association between rodent diversity and deermice encounter rates in Utah. The different species composition of rodent assemblages between sites (Utah vs Chihuahua) likely results in differences in interspecific, and perhaps even intraspecific interactions among individual rodents in the assemblage. Differences may also be attributed to different research methods; the investigators in Utah did not experimentally manipulate the rodent community, therefore, density and species diversity were not controlled. On the other hand, our experiment, which comprised an analysis at a small spatial scale and a reduced number of individuals and species, may not capture all possible interactions among rodents in natural field conditions. However, because the species used in our experiment account for nearly 85% of the total abundance of rodents in our area [19], the three non-competent host species are likely to interact more with deermice than other rodent species in the community. We also highlight that the presence of the non-competent hosts did not influence the frequency of visits of deermice to foraging stations, which can influence intraspecific encounter rates.

In our system, we found a high heterogeneity in the frequency of interspecific interactions. Kangaroo rats had significantly more interactions with deermice, and such interactions were in most cases aggressive (kangaroo rats often attacked and chased deermice, and deermice often avoided kangaroo rats). Similarly, Falkenberg and Clarke [36] found that deermice avoid encounters with Ord's Kangaroo rats (*Dipodomys ordii*) even changing their microhabitat to promote separation. Clay et al. [15] suggested that Ord's Kangaroo rats may increase prevalence of SNV in deermice in that its presence can concentrate deermice individuals, which in turn would increase contact rates among deermice. In our experiment, we did not find an effect of kangaroo rats on the frequency of interactions between deermice. A possible explanation is that we only assessed encounters at foraging stations. Also, the two species had different activity patterns, which could reduce their interactions. In fact, according to our results, we suggest that deermice change their activity pattern to avoid encounters with kangaroo rats. On the other hand, pocket mice and grasshopper mice had very few interactions with deermice. For example, although grasshopper mice (*Onychomys* spp.) can eat grains and seeds, they feed primarily on insects, which may be a reason for the low frequency of interactions with deermice at the foraging stations. Also, the fact that grasshopper mice occasionally prey on other rodent species [37,38] could trigger avoidance behaviors by other rodent species and potentially reduce interactions among deermice individuals. However, our experiments fail to evince such a reduction, in fact, deermice showed aggressive behavior toward grasshopper mice in some interaction events. Both grasshopper mice and pocket mice are similar to deermice in size, whereas kangaroo rats are bigger. Therefore, the size of potential rodent competitors may determine the behavioral response of deermice to interspecific encounters.

### Temporal activity patterns and their relevance when analyzing interactions between hosts

Increasing evidence has demonstrated the ecological significance of time use to understand relationships among species in communities at local scales [39]. However, patterns of temporal activity of rodent assemblages have been scantly studied [40]. Because diel activity patterns may influence interactions among species, they can indirectly affect pathogen transmission. In our experimental system, all rodent species had a similar time activity range; consequently, a high temporal niche overlap existed at the whole assemblage level, thus providing evidence that all species have similar limitations in the way they exploit their environment [39]. Other studies have found that small mammal assemblages from other ecosystems can exhibit high temporal niche segregation [40]. This segregation should imply few interactions between species that do not share the same patterns of temporal activity. These differences in how species within an assemblage use time demonstrate that this little studied topic can have profound implications for pathogen transmission dynamics based on the differences in assemblage composition. Although we reported a high temporal activity overlap of rodent species in our study, we found differences in activity patterns between some species, as well as differences in the activity of each species between seasons. More interesting, when we separated the analysis by season, the activity patterns of deermice changed in the presence of kangaroo rats. These findings indicate that the effect of a particular non-competent hosts species on the activity patterns of deermice may be more important than the effect of species richness *per se* on activity patterns of the focal species. Even though we did not find an effect of kangaroo rats or species richness on the number intraspecific interactions of deermice, given that there was an effect of season on activity patterns for all species analyzed, a large number of replicates of the experiments through different seasons should be conducted for further evaluation of the effect of non-competent hosts on intraspecific interactions of deermice.

### Limitations of the experiment

We acknowledge the limitations of this study in that we use three enclosures to conduct six experimental trials of each of three treatments. In an ideal study design, there would be more replicates of enclosures to meet the assumptions of independence in statistical tests. Unfortunately, more enclosures require a large area and much expensive construction material. To reduce possible bias due to eventual differences between enclosures that could influence host behaviors, we alternated the enclosure housings for each treatment, therefore, each enclosure was used for two trials of each treatment.

Another limitation was that we only monitored rodent interactions at foraging stations. Interactions during foraging are possible opportunities for hantavirus transmission, as deermice can fight over food resources [34,41]. However, future experiments should also include other monitoring areas within the enclosures, for example, placing cameras near burrows, as there may be competition of rodents for burrows.

Deermice used in this experiment were not tested for hantavirus infection prior to the experiment. Deermice with bold behavior (e.g. aggressive) are more likely to be infected with SNV than shy deermice [34]. The infection with SNV is more likely to be the consequence of increased encounters (it is less likely that increased encounters are the result of infection [34,42]). As we randomly placed deermouse individuals into the different enclosures, we believe that we avoided, as much as possible, a bias of including a pool of deermice infected in a particular treatment or trial. However, future experiments should also take into account hantavirus infection in individuals used in the experiments.

### Future studies need to account for variation in deermice density and seasons

In directly transmitted pathogens, a dilution effect can occur, for example, if increased host richness leads to a reduction in abundance of competent hosts (susceptible host regulation) or, on the other hand, leads to a reduction of interaction rates among competent hosts regardless of host abundance (transmission interference) [3]. Using longitudinal datasets of long-term surveys of SNV infection in deermice across the southwestern US, Luis and Mills [43] conducted a Susceptible-Infected epidemiological model and found that transmission was density-dependent and that increased small mammal richness leads to decreased deermouse population density, which in turn, caused a decline in SNV antibody prevalence. Similarly, Suzán et al. [17] found that increasing rodent species richness in Panama was associated with a decrease in hantavirus reservoir abundance and a decrease in hantavirus antibody prevalence. Both studies suggest that the mechanism behind the dilution effect in those systems is “susceptible host regulation”. In our study, we maintained deermice population density constant across treatments to allow the assessment of any potential effects of rodent species richness *per se* on deermice interactions. Therefore, future investigations need to include a systematic change in deermice population density along with a greater variation in rodent species richness.

Host-pathogen systems often have seasonal variation in transmission (44). Several factors that change seasonally can influence disease dynamics, for example, precipitation, resource availability, reproductive activity of hosts, etc. [44]. In fact, hantaviruses can have seasonal cycles of infection prevalence [11,45,46]. As mentioned above, our results showed that rodents shift their activity patterns among seasons. This finding can be relevant for shaping rodent interactions and, in turn, for transmission of hantaviruses. In addition, transmission dynamics of some directly transmitted pathogens may vary between density dependence and frequency dependence according to season [47] and deermouse population density also varies seasonally [26]. Therefore, the inclusion of seasonal variation in new studies can provide further insights into deermice behavior and interactions that may have implications for hantavirus transmission.

### Concluding remarks

In our study system, intraspecific interactions of deermice during foraging seemed not to be influenced by other dominant species of the rodent assemblage. Thus, a potential dilution effect in this deermouse-hantavirus system would not be through intraspecific encounter reduction in the most competent hantavirus host. We observed that a non-competent host species influenced deermice activity patterns and interspecific behavior. Therefore, more investigations are needed to assess the effect of non-competent hosts on intraspecific interactions of deermice. These studies should consider the recommendations we provide above, including a larger number of replicates of each treatment through seasons, placing cameras in other areas of the enclosures, and treatments with varying densities of deermice.

The dilution effect hypothesis is under debate [48,49]. A critique of the hypothesis is that composition of species in communities may be more important than simple measures of species richness or diversity [50]. Pathogen transmission might increase or decrease in high-diversity communities due to the increased chance of including a particular species, a phenomenon called “identity effect” [51,52]. Therefore, the identity effect may play an important role in the diversity–disease relationship (reviewed by Huang et al. [48]). Our findings highlight the need to address the identity of species and the composition of the communities that can influence both interactions and abundances of competent hosts.

There is a consensus for the need to investigate the mechanisms that drive dilution or amplification effects for moving forward with research on the biodiversity-disease relationship [2,53]. To our knowledge, this study represents the first manipulative experiment in outdoor enclosures to assess the effect of species richness on interactions and temporal activity patterns among hosts. This approach offers the potential to shed further light on the mechanisms by which species diversity and community composition influence disease transmission.

## Supporting information

S1 Table Pairwise comparisons (Watson's U^2^ test) of activity patterns among rodents combining both seasons. Values above the diagonal correspond to P values. Values below the diagonal dashes correspond to U^2^ statistic values. Significant results in bold.
(DOCX)

S2 Table Results of ROSARIO algorithm null model analyses of temporal niche overlap (analyses separated by seasons). Overlap was quantified as the average of all pair-wise overlap values calculated via the Czechanowski index, using the numbers of records for each species at two time intervals (30 min and 1 h). P-values are two-tailed probabilities of finding non-random assemblage-wide temporal niche overlap. Tail (T) indicates if empirical overlap occurs on the left-hand (L) or right-hand (R) side of the simulated distribution. Values on the extreme left would have indicated segregated activities and those on the extreme right coincident activity patterns. Significant results in bold.
(DOCX)

S1 Dataset Intra- and interspecific interactions of deermice and time of day of each visit to the foraging stations by each species. (XLSX)
