# Supplementary material for: Is species richness driving intra- and interspecific interactions and temporal activity overlap of a hantavirus host? An experimental test
Source: PLoS One. 2017 Nov 15;12(11):e0188060. doi: 10.1371/journal.pone.0188060 (PMC5687724; doi:10.1371/journal.pone.0188060)
Supplement: S1 Table — Values above the diagonal correspond to P values. Values below the diagonal dashes correspond to U2 statistic values. Significant results in bold. (DOCX) [file pone.0188060.s001.docx]

**S1 Table**

| **Deermouse^1^** | **T1** | **T2** | **T3** |  |
| --- | --- | --- | --- | --- |
| **T1** | - | 0.1 > p > 0.05 | 0.5 > p > 0.2 |  |
| **T2** | 0.16 | - | 0.1 > p > 0.05 | |
| **T3** | 0.07 | 0.19 | - |  |
| **Interspecies** | **Deermouse** | **Kangaroo rat** | **Pocket mouse** | **Grasshopper mouse** |
| **Deermouse** | - | **< 0.001** | **< 0.001** | 0.5 > p > 0.2 |
| **Kangaroo rat** | 2.7 | - | **< 0.01** | **< 0.001** |
| **Pocket mouse** | 0.9 | 0.27 | - | **< 0.02** |
| **Grasshopper mouse** | 0.11 | 0.64 | 0.27 | - |

^1^ Comparison of deermice between treatments (T).
