# Supplementary material for: Is species richness driving intra- and interspecific interactions and temporal activity overlap of a hantavirus host? An experimental test
Source: PLoS One. 2017 Nov 15;12(11):e0188060. doi: 10.1371/journal.pone.0188060 (PMC5687724; doi:10.1371/journal.pone.0188060)
Supplement: S2 Table — Overlap was quantified as the average of all pair-wise overlap values calculated via the Czechanowski index, using the numbers of records for each species at two time intervals (30 min and 1 h). P-values are two-tailed probabilities of finding non-random assemblage-wide temporal niche overlap. Tail (T) indicates if empirical overlap occurs on the left-hand (L) or right-hand (R) side of the simulated distribution. Values on the extreme left would have indicated segregated activities and those on the extreme right coincident activity patterns. Significant results in bold. (DOCX) [file pone.0188060.s002.docx]

**S2 Table.**

|  |  | **Rosario** | | | |
| --- | --- | --- | --- | --- | --- |
| **Tested group** | **Observed**  **overlap** | **Simulation**  **overlap** | **SD** | **P-value** | **T** |
| **Late Winter** |  |  |  |  |  |
| *30-min interval* |  |  |  |  |  |
| Treatment 3^1^ | 0.80 | 0.70 | 0.025 | **0.025** | R |
| All species^2^ | 0.79 | 0.68 | 0.02 | **0.002** | R |
|  |  |  |  |  |  |
| *1-h interval* |  |  |  |  |  |
| Treatment 3^1^ | 0.80 | 0.68 | 0.026 | **0.008** | R |
| All species^2^ | 0.82 | 0.68 | 0.02 | **< 0.001** | R |
| **Spring** |  |  |  |  |  |
| *30-min interval* |  |  |  |  |  |
| Treatment 3^1^ | 0.83 | 0.68 | 0.04 | 0.07* | R |
| All species^2^ | 0.82 | 0.70 | 0.02 | **0.003** | R |
|  |  |  |  |  |  |
| *1-h interval* |  |  |  |  |  |
| Treatment 3^1^ | 0.87 | 0.68 | 0.03 | **0.03** | R |
| All species^2^ | 0.87 | 0.66 | 0.03 | **0.001** | R |

^1^All three species from treatment 3.

^2^All four species used in the experiment. Data of deermice from all treatments were pooled.

*marginally significant
